# Diversities of Sexual Activities and Correlates of Safe Sex Practices Among Adolescents in Plateau State, Nigeria

**DOI:** 10.3389/frph.2021.744622

**Published:** 2021-11-12

**Authors:** Esther Awazzi Envuladu, Karlijn Massar, John B. F. de Wit

**Affiliations:** ^1^Department of Community Medicine, College of Health Sciences, University of Jos, Jos, Nigeria; ^2^Department of Interdisciplinary Social Science, Utrecht University, Utrecht, Netherlands; ^3^Department of Work and Social Psychology, Maastricht University, Maastricht, Netherlands

**Keywords:** adolescent, sexual diversity, consistent condom use, Plateau State, Nigeria

## Abstract

**Background:** Unsafe sex, particularly, condomless sex exposes adolescents to sexual and reproductive health risks. This study aimed to assess the sexual experiences and to determine the most important covariates of sexual activity and consistent condom use among adolescents in Plateau State, Nigeria.

**Methods:** A cross sectional survey was conducted among 428 adolescents selected from 6 LGAs through a multistage sampling technique. The data was analyzed using the IBM Statistical package for Social Sciences (SPSS) version 23, multiple logistic regression was conducted to determine the covariates of sexual activity and condom use.

**Results:** About one third (38%) of the adolescents were sexually active, 5.7% had same sex partners, 70% had more than one sexual partner and majority (72.4%) were not consistently using condom during sex. Logistic regression results showed that older adolescents (OR = 5.73; CI = 3.72–8.12; *p* = 0.001) and out of school adolescents (OR = 2.68; CI = 1.79–4.00; *p* = 0.001) were more likely to be sexually active, while multivariable logistic regression analysis showed age (AOR = 0.33; CI = 0.12–0.90; *p* = 0.031) and gender as important covariates of being sexually active, (AOR = 6.29; CI = 3.18–12.44; *p* = 0.001). Inconsistent condom use was more likely among adolescents; with lower education, (OR = 2.14; CI = 1.19–3.85; *p* = 0.011), having sex with older partners (OR = 0.61; CI = 0.42–0.90; *P* = 0.013) and with low awareness of SRH issues (OR = 2.08; CI = 1.02–4.22; *p* = 0.044). The multivariable logistic regression however, showed gender, being male (AOR = 0.43; CI = 0.006–3.09; *p* = 0.023) as covariate of consistent condom use.

**Conclusion:** Most sexually active adolescents had multiple sexual partners, some had same sex partners and majority were not consistently using condom. Older adolescents and those out of school were more likely to be sexually active. Awareness of SRH issues significantly influenced condom use while gender, specifically being male, was the independent covariate for being sexually active and for consistent condom use. We recommend sexual health intervention targeted at adolescents. In addition, gender should be mainstreamed into adolescent sexual and reproductive health programmes.

## Introduction

Adolescence is a period of major developmental transitions, marked by physiological, physical and emotional changes, and the acquisition of advanced impulse regulation (1). Together, these changes have a profound influence on adolescent's decision making, risk taking, and social relationships (1). Adolescence is also a critical period for romantic and sexual development, and across the world, adolescents are known to become sexually active and gain diverse sexual experiences (2). As elsewhere, a significant proportion of adolescents in Nigeria are sexually active, and the sexual conduct of adolescents in Nigeria has similarly attracted public health concerns, particularly because of the high prevalence of unsafe sex, mainly condomless sex among this age group (3).

Sex without a condom is the major unsafe practice which exposes adolescents to various risks, such as unintended pregnancy, early child birth and unsafe abortion, and also puts them at a high risk of contracting sexually transmitted infections (STIs), including HIV (4, 5). Adolescent pregnancy is a public health challenge in Africa, and adolescent pregnancies are one of the major reasons for the high rate of maternal mortality in Nigeria (6). A systematic review and meta-analysis estimated a pooled adolescent pregnancy rate of 18.8% in Africa, 19.3% in Sub-Saharan African, and 15.9% in Nigeria (6). Recent data has shown an upward trajectory in the rates of STIs, notably gonorrhea, chlamydia, and human papilloma virus infection among adolescents and young people (7). While the HIV epidemic appears to be decreasing in Nigeria and other African countries, this gain is limited and there is evidence that HIV prevalence is still high among adolescents and young people and AIDS remains a leading cause of mortality among young people in Africa (7, 8).

Condom use, which is one of the most cost effective protective measures against unintended pregnancy and STI/HIV is underpinned by complex factors. Research has found that social, economic and cultural factors are related to condomless sex (9–13). Gender dynamics are important drivers of non-condom use because of the inability of both partners to openly communicate safe sex, due to the power differences that exist between males and females in Africa (14). Also, early sexual initiation, having multiple sexual partners and intergenerational sex, that is, a sexual relationship with an older partner are factors found to be associated with inconsistent condom use among adolescents in some studies (15, 16). In many cases, sex with older partners is transactional, in exchange for material, money and other benefits. In Nigeria, transactional sex is a common practice among adolescent girls as well as boys, and this poses a challenge for adolescents to negotiate protective options such as consistent (i.e., at every sexual intercourse) condom use (9, 17, 18). Aside the challenge adolescents have negotiating condom use with older partners; consistent condom use is linked to adolescent's (lack of) awareness/knowledge of SRH issues, and risk perception regarding STIs, HIV, and pregnancy (19–21). Research in several sub-Sahara African countries (e.g., Nigeria, South-Africa, Zimbabwe, Mozambique) found that adolescent's low perceived risk of HIV or other STIs contributes significantly to their likelihood of not (consistently) using a condom during sexual intercourse (22–25).

The risk perception may also be due to lack of adequate knowledge on sexual and reproductive health matters subsequent to poor communication between parents and adolescents on sexuality issues (26). Like in many African countries, adolescent sexual behavior is a sensitive matter in the context of dominant religion and culture (27, 28).

Parents in Nigeria sometimes deny adolescent involvement in sex outside marriage and typically adhere to strict, traditional discipline in the upbringing of their children that makes it difficult to accept that adolescents may have sex (29). In contrast, most adolescents are exposed to information about sex through the Internet (27), and frequently engage in (unsafe) sex (2, 27). Although the Nigerian government made a commitment to provide ASRH services to address adolescent's sexual and reproductive health issues, these services are often not available–and when they are, they are of low quality and do not meet adolescent's needs (30).

As most young people become sexually active in adolescence, including in Nigeria, it is important to prevent adverse outcomes and promote the sexual health and well-being of adolescents. This study therefore aimed to assess the sexual experiences of adolescents in Plateau State, Nigeria, and to examine socio-demographic characteristics and awareness of SRH issues, knowledge of prevention of SRH problems, and perceived risk of SRH problems as covariates of their sexual activity and safe sex practices, specifically consistent condom use. The findings from this study may inform governmental policy development and decision-making regarding the provision and implementation of adolescent sexual and reproductive health care services across Nigeria.

## Methods

### Study Design and Setting

A cross sectional survey was conducted among adolescents 18–19 years who resided in Plateau State, one of the 36 States in Nigeria, located in the central part of the country. Plateau State has an estimated population of 4,376,193, of which about 23% are adolescents. In Nigeria, states are broadly divided into 3 senatorial zones and geographically divided into local government areas (LGA), the LGAs are further divided into political wards which is also recognized by WHO. There are 17 LGA in Plateau State, and three senatorial zones; the Northern senatorial zone has six LGAs, the Central zone has five LGAs, and the Southern zone consists of six LGAs. The current study was conducted in 6 LGAs, two LGAs in each of the three senatorial zones.

### Participants and Recruitment

The sample size was determined using the Cochran formula (*n*_0_ = Z^2^pq/e^2^), (31) where n is the minimum sample size, Z is the standard normal deviate which corresponds to a 95% confidence interval (1.96), p is the proportion of adolescents who are sexually active, here assumed to be 50%, q is the complimentary probability (1-p), and e is the 5% margin of error. A minimum sample size of 384 was determined based on this calculation.

Participants were recruited through a multistage sampling technique. First, two LGAs were selected using a simple random sampling technique by balloting from the list of LGAs in each of the senatorial zones, giving a total of 6 LGAs. Next, from each of these six LGAs, three wards were randomly selected from approximately 15 wards in each LGA by balloting. At the ward level, the research team collaborated with community mobilizers (i.e., local government staff that serve as community liaison officers) and youth leaders (i.e., the official representatives of organized youth groups in each ward) to identify houses with adolescents aged 18–19 years that could participate in the study, total sampling of all the houses with eligible adolescents was done. A team of research assistants who were medical students aged 19–22 years collected the data by going from house to house and asking eligible adolescents who gave consent to participate. The research assistants received training in data collection for the study, which included practical demonstrations and undertaking pretesting of the questionnaire in a different community setting before the actual data collection. In total, 1,008 houses were visited. Where more than one adolescent was present in the household, balloting was used to select one adolescent. A total of n = 479 adolescents gave consent and participated in the study; 428 questionnaires were completely filled out and eligible for inclusion in the analyses.

### Measures

*Socio-demographic* characteristics included age, gender, marital status, schooling status (in school or out of school) and highest level of educational qualification.

Diverse sexual experiences of adolescents assessed included being in an intimate relationship, being sexually active (i.e., currently having sex with someone), those sexually active were asked age at sexual debut; gender of sexual partner(s) i.e. same sex or opposite sex, age of sexual partner(s), marital status of sexual partner(s), and number of sexual partners.

*Consistent condom use* during sex was specifically asked among those who were sexually active by asking if condom was used at every sexual intercourse. Those who indicated to always use condom were considered as consistent condom use while those who responded to never or not always using a condom were considered inconsistent condom use.

*Awareness of SRH* issues were assessed by three questions, with responses indicated as yes (scored 1) or no (scored 0): “Have you heard about sexual and reproductive health?” “Have you heard about STI?” and “Have you heard about HIV?” The total scores for level of awareness ranged from 0 to 3.

*Knowledge of prevention of SRH problems* was assessed by asking if they knew how to prevent pregnancy, STI and HIV, those who answered yes to any of the three questions were further asked to mention specific methods of prevention of pregnancy, STI and HIV. Appropriate responses (i.e., the use of condom for prevention of pregnancy, STI and HIV or the use of contraceptives for prevention of pregnancy) was scored 1 each, whereas those who gave inappropriate responses were scored 0; total scores ranged from 0 to 3. Those who scored ≤1 were considered to have poor knowledge, those who scored 2 were considered to have fair knowledge, and those who scored 3 were considered to have good knowledge.

*Perceived risk* of becoming pregnant, becoming infected with STI or with HIV during condomless sex was assessed with three questions: “Do you think you are at risk of pregnancy during unprotected sex?” “Do you think you are at risk of sexually transmitted infection during unprotected sex?” and “Do you think you are at risk of HIV infection during unprotected sex?” Total scores ranged from 0 to 3, those who responded that they were not at risk during unprotected sex were scored 0 while those who responded that they were at risk during unprotected sex were scored 1. The scores of 0 or 1 were considered low risk perception, whereas scores of 2 or 3 were considered high risk perception.

### Data Analysis

Frequency analysis was used to describe the socio-demographic characteristics of adolescents, as well as their diverse sexual experiences, their awareness of SRH issues, knowledge of prevention of SRH problems, and perceived risk of SRH problems. Univariate and multivariable logistic regression analyses were conducted to assess covariates of sexual activity and consistent condom use. The data were analyzed using the IBM Statistical package for Social Sciences (SPSS), version 23.

### Ethical Approval

Ethical clearance was obtained from the Jos University Teaching Hospital (JUTH) Human and Research Ethics Committee, and permission was obtained from the Plateau State Ministry of Health, the selected LGA authorities and the village heads of the various communities in the selected wards before commencement of the study. Written informed consent was obtained from the participants after a detailed explanation of the study.

## Results

### Participant Characteristics

Slightly more males than females participated in the study (51.9% males and 48.1% females) 52.3% were 18 years old and 47.7% were 19 years; 5.8% of the respondents were married adolescents and 41.1% of the respondents were out of school at the time of the study while 11.7% of the adolescents had no formal education or just a primary level of education (see Table 1).

**Table 1:** Socio-demographic characteristics of participating adolescents in Plateau State, Nigeria (*N* = 428).

**Socio-demographic characteristics**	**Frequency**	**%**
**Gender**		
Male	222	51.9
Female	206	48.1
**Age in years**		
18	224	52.3
19	204	47.7
**Marital status**		
Married	25	5.8
Not married	403	94.2
**Religion**		
Christianity	357	83.4
Islam	71	16.6
**Schooling status**		
In school	252	58.9
Out of school	176	41.1
**Educational level**		
No formal/primary education	50	11.7
Secondary education	317	74
Tertiary education	61	14.3

### Sexual Diversities and Sexual Risk Behavior

More than half of the respondents (57.2%) indicated they were in an intimate relationship and 38.1% were sexually active at the time of the study. Respondent's age at sexual debut ranged from 6 to 19 years, and the mean age at sexual debut was 15.9 years (SD = 2.94). The majority had sexual partners of the opposite sex, while 5.7% (also) had same-sex partners. About 70% had more than one sexual partner, 40% had 2–3 sexual partners, and 15.3% had more than 5 sexual partners. Majority (72.4%) of the sexually active adolescents were not consistently using condom during sex. and most respondent's (61.3%) had low risk perception, just about half had good awareness of SRH issues (51.5%), and knowledge of prevention of SRH problems (58.9%) (see Table 2).

**Table 2:** Sexual experience, risk perceptions and awareness of SRH among adolescents in Plateau State, Nigeria.

**Variables**	**Frequency**	**Percentage**
**In intimate relationship** **(***N*** = 428)**		
Yes	245	57.2
No	183	42.8
**Gender of intimate partner** **(***N*** = 245)**		
Opposite sex	231	94.3
Same sex	14	5.7
**Currently Sexually active (245)**		
Yes	163	38.1
No	265	61.9
**Age at first sexual intercourse(163)**		
<10–13 years	23	14.1
14–16 years	53	32.5
17–19 years	87	53.4
**Condom use (163)**		
Inconsistent	118	72.4
Consistent	45	27.6
**Risk perception** **(***N*** = 163)**		
Low risk	100	61.3
High risk	63	38.7
**Awareness of SRH issues**		
low awareness	79	48.5
high awareness	84	51.5
**Knowledge of prevention of SRH problems**		
Poor knowledge	67	41.1
Good knowledge	96	58.9

### Co-variates of Being Sexually Active

As shown in Table 3, univariate logistic regression analyses showed that older adolescents were more likely to be sexually active than the younger adolescents (OR = 5.73; CI = 3.72–8.12; *p* = 0.001), those out of school were more likely to be sexually active than those in school (OR = 2.68; CI = 1.79–4.00; *p* = 0.001), and those who were not in any intimate relationship were unlikely to be sexually active (OR = 0.14; CI = 0.09–2.2; *p* = 0.001). Multivariable logistic regression analysis showed that male gender (AOR = 0.33; CI = 0.12–0.90; *p* = 0.031), older age (AOR = 6.29; CI = 3.18–12.44; *p* = 0.001) and being in an intimate relationship (AOR = 0.08; CI = 0.03–0.23; *p* = 0.001) were covariate of being sexually active, schooling status (*p* = 0.580) and educational level (*p* = 0.642) were however not found to be significant covariates of being sexually active.

**Table 3:** Covariates of being sexually active among adolescents in Plateau State, Nigeria (*N* = 428).

	**Sexually active**							
	**Yes**	**No**	**OR**	**95% CI**	**p**	**AOR**	**95% CI**	***p***
**Gender**								
Male	85(38.3)	137(61.7)	0.98	0.67–1.45	0.928	0.33	0.12–0.90	0.031
Female	78(37.9)	128(62.1)						
**Age**								
18 years	44(19.6)	180(80.4)	5.73	3.72–8.12	0.001	6.29	3.18–12.44	0.001
19 years	119(58.3)	85(41.7)						
**Marital status**								
Married	16(64.0)	9(36.0)	0.32	0.14–0.75	0.008	0.21	0.06–0.81	0.023
Not married	147(36.5)	256(63.5)						
**Schooling status**								
In school	72(28.6)	180(71.4)	2.68	1.79–4.00	0.001	1.21	0.62–2.36	0.58
Not in school	91(51.7)	85(48.3)						
**Education level**								
No formal or primary education	27(54.0)	23(46.0)	1.25	0.85–1.84	0.256	1.11	0.71–1.74	0.642
Secondary education	99(31.2)	218(68.8)						
Tertiary education	37(60.7)	24(39.3)						
**In intimate relationship**								
Yes	136(55.5)	109(44.5)	0.14	0.09–2.2	0.001	0.08	0.03–0.23	0.001
No	27(14.8)	156(85.2)						

### Covariates of Consistent Condom Use

As shown in Table 4, univariate logistic regression analyses showed that males were more likely to consistently use condoms than females (OR = 0.44; CI = 0.21–0.90; *p* = 0.024), those with no education/primary education were more unlikely to consistently use condoms compare to those with higher levels of education (OR = 2.14; CI = 1.19–3.85; *p* = 0.011), and those with sexual partners within their age group were more likely to consistently use condom compare to those with older sexual partners (OR = 0.61; CI = 0.42–0.90; *P* = 0.013). Also, participants with higher level of awareness of SRH issues were more likely to consistently use condoms than those with lower level of awareness of SRH issues (OR = 2.08; CI = 1.02–4.22; *p* = 0.044). The multivariable logistic regression analysis however showed male gender (AOR = 0.43; CI = 0.006–3.09; *p* = 0.023) as the only significant covariate of consistent condom use while age (*p* = 0.843), education (*p* = 0.102), age of sexual partners (*p* = 0.142) and number of sexual partners (*p* = 0.325) were not covariate of consistent condom use.

**Table 4:** Covariates of consistent condom use among sexually active adolescents in Plateau State (*N* = 163).

**Demographic characteristics**	**Use of condom**							
	**Inconsistent**	**Consistent**	**OR**	**95%C. I**.	***P***	**AOR**	**95% C. I**.	***P***
**Age**								
18	33(75.0)	11(25.0)	1.2	0.55–2.64	0.651	0.89	0.29–2.78	0.843
19	85(71.4)	34(28.6)						
**Gender**								
Male	55(64.7)	30(35.3)	0.44	0.21–0.90	0.024	0.43	0.06–3.09	0.023
Female	63(80.8)	15(19.2)						
**School status**								
In school	48(66.7)	24(33.3)	0.6	0.30–1.20	0.148	1.1	0.37–331	0.865
Out of school	70(76.9)	21(23.1)						
**Education**								
No formal/Primary	25(92.6)	2(7.4)	2.14	1.19–3.85	0.011	2.39	0.84–6.80	0.102
Secondary	70(70.7)	29(29.3)						
Tertiary	23(62.2)	14(37.8)						
**Age at first sex**								
18	99(70.7)	41(29.3)	0.51	0.16–1.59	0.244	0.61	0.13–3.01	0.866
19	19(82.6)	4(17.4)						
**Age of sexual partner**								
≤19	37(63.8)	21(36.2)	0.61	0.42–0.90	0.013	0.59	0.29–1.20	0.142
20–29	47(71.2)	19(28.8)						
30–39	10(83.3)	2(16.7)						
≥40	24(88.9)	3(11.1)						
**Marital status of sexual partner**								
Single	82(70.7)	34(29.3)	0.74	0.34–1.62	0.446	3.79	0.87–16.49	0.076
Married	36(76.6)	11(23.4)						
**Number of sexual partners**								
One	38(79.2)	10(20.8)	0.97	0.69–1.36	0.863	0.76	0.44–1.32	0.325
Multiple	80(69.6)	35(30.4)						
**Awareness of SRH**								
Low awareness	63(79.7)	16(20.3)	2.08	1.02–4.22	0.044	1	0.10–9.72	0.998
High awareness	55(65.5)	29(34.5)						
**Knowledge of Prevention**								
Poor knowledge	67(100.0)	0(0.0)	–	–	–	–	–	–
Good knowledge	51(53.1)	45(46.9)						
**Risk perception**								
Low risk	63(100.0)	0(0.0)	–	–	–	–	–	–
High risk	55(55.0)	45(45.0)						

*OR for the last two variables cannot be computed because condom consistency is zero*.

## Discussion

Young people in Nigeria are at risk of several sexual and reproductive health problems such as unintended pregnancy, STIs, and HIV (6). Therefore, the aims of the current study were to assess the sexual experiences of adolescent boys and girls in Plateau State, Nigeria, to understand the factors that influence their sexual activity, and to determine the most significant covariates of (in)consistent condom use. The latter is an important contributing factor for mitigating the health risks these young people are exposed to.

Our results show that although the majority had sexual partners of the opposite sex, few of the adolescents had same sex partners. In addition, more than three quarter of the sexually active adolescents were not consistently using condoms during sex. This is especially concerning because more than half of our respondents indicated a low risk perception for unintended pregnancies or disease transmission. Further, almost half of the adolescents had a low level of awareness of SRH issues and poor knowledge of how to prevent pregnancy, STI and HIV. These findings are corroborated by similar studies, for example, a meta-analysis of research in sub-Saharan African countries and Nigeria reported high rates of sexual activity and multiple sexual partners among adolescents males and females, as well as a large age difference between adolescents and their sexual partners (32–34). Also, studies conducted among adolescents in Nigeria have reported inconsistent use of condoms during sex, as well as a low risk perception and lack of knowledge of SRH issues and how to prevent SRH problems (11, 12, 22).

Our results show that older adolescents were five times likely to be sexually active than younger ones, adolescents out of school were twice as likely to be sexually active than those in school, and males were more likely to be sexually active than females. This is in line with studies conducted in the western and Eastern part of Nigeria (35, 36). Moreover, gender (i.e., being male) was an important factor linked to the sexual activity of adolescents in three consecutive demographic and health surveys in Nigeria (34). Some studies have also linked the role of gender in sexual activity especially in Africa to the patriarchal norms; where males are seen as superior to females and permitted by society to always have their ways, this dominant role exert influence on their sexual behavior (1, 37).

Factors found to be associated with inconsistent condom use in this study were a lower level of education and having sex with older partners: Adolescents with a lower level of education were twice as likely to have inconsistent condom use during sex compared to those with a higher education, while those who reported to have sexual partners within their own age group were more likely to consistently use a condom during sex. Similarly, some studies have documented inconsistent condom use among adolescents in relationship with older partners; the younger partners usually find it difficult to persuade the older partners especially when the partners are unwilling to use condom (37–39). However, the one unique covariate of consistent condom use in this study is gender; the adolescent males were more likely to consistently use condom during sex than the female adolescents. Similar to our finding is the results from studies in some African countries and across Nigeria, which also reported more condomless sex among females, attributing it to gender inequality which does not encourage free dialogue and communication between partners (37, 40–42).

One of the strength of this study is the privacy and confidentiality provided during data collection. Specifically, were the research assistances that were their age group that gave the respondents the freedom to provide information on their sexual life more freely. Furthermore, the diversity of the adolescents (males and females, in-school and out of school, lower education and higher education) in this study gave a broader perspective of the sexual experiences and the factors influencing condom use among adolescents. The study however did not capture the younger adolescents, mainly due to ethical reasons around consent when conducting studies among younger adolescents, thereby limiting our understanding of the particular experiences and challenges of this younger group of adolescents. We are also not ruling out recall bias from self-reporting and social desirability bias in reporting sexual activity though they were asked to be honest in their responses and assured of confidentiality.

## Conclusion and Recommendations

In this study, we found that a good number of adolescents were sexually active with diverse experiences. Both male and female adolescents had multiple sexual partners and some had same sex partners. The majority of the sexually active adolescents had low risk perception and were not consistently using condom during sex. We also found that more than half of the adolescents had low risk perception of getting pregnant/becoming infected with STI or HIV during unprotected sex and about half were not aware of SRH issues. Gender was seen to significantly influence safe sex, specifically being male, was the independent covariate for being sexually active and for consistent condom use.We recommend that more sexual health promotion activities geared toward safe sex practices be targeted at both in school and out of school adolescents. In addition, gender should be mainstreamed into ASRH programmes to ensure a better and healthier sexual experience among adolescents.

## Data Availability Statement

The raw data supporting the conclusions of this article will be made available by the authors, without undue reservation.

## Ethics Statement

The studies involving human participants were reviewed and approved by Jos University Teaching Hospital (JUTH) Research Ethics Committee. The patients/participants provided their written informed consent to participate in this study.

## Author Contributions

All authors listed have made a substantial, direct and intellectual contribution to the work, and approved it for publication.

## Conflict of Interest

The authors declare that the research was conducted in the absence of any commercial or financial relationships that could be construed as a potential conflict of interest.

## Publisher's Note

All claims expressed in this article are solely those of the authors and do not necessarily represent those of their affiliated organizations, or those of the publisher, the editors and the reviewers. Any product that may be evaluated in this article, or claim that may be made by its manufacturer, is not guaranteed or endorsed by the publisher.
